# The burden of scrub typhus in India: A systematic review

**DOI:** 10.1371/journal.pntd.0009619

**Published:** 2021-07-27

**Authors:** Emily Devasagayam, Divya Dayanand, Debasree Kundu, Mohan S. Kamath, Richard Kirubakaran, George M. Varghese

**Affiliations:** 1 Department of Infectious Diseases, Christian Medical College, Vellore, Tamil Nadu, India; 2 Department of Reproductive Medicine, Christian Medical College, Vellore, Tamil Nadu, India; 3 South Asian Cochrane Network and Centre, Christian Medical College, Vellore, Tamil Nadu, India; SCI Foundation, AUSTRALIA

## Abstract

**Background:**

Scrub typhus, a vector-borne zoonotic infection caused by the bacteria *Orientia tsutsugamushi*, is one of the most common and clinically important rickettsial infections worldwide. An estimated one million cases occur annually with a high case fatality rate. Although scrub typhus is a major public health threat in India, the burden and distribution remains unclear. We aimed to estimate the burden of scrub typhus in India.

**Methodology:**

We performed a systematic review of published literature on scrub typhus from India to extract information on epidemiology, morbidity, and mortality. Important databases were searched using keywords and appropriate combinations. We identified observational, interventional, and population-based studies and extracted the data to evaluate the number of cases diagnosed using serology or PCR and the number of deaths due to scrub typhus. We conducted a systematic narrative synthesis to summarize included studies.

**Principal findings:**

In the last decade, there were 18,781 confirmed scrub typhus cases reported in 138 hospital-based studies and two community-based studies. IgM ELISA was used in 122 studies to confirm the cases in majority (89%). The proportion of scrub typhus among acute undifferentiated febrile illness (AUFI) studies was 25.3%, and community seroprevalence was 34.2%. Ninety studies had data published on multiple organ involvement out of which 17.4% of cases had multiple organ dysfunction syndromes, 20.4% patients required ICU admission, and 19.1% needed ventilation. The overall case-fatality rate was 6.3%, and the mortality among those with multi-organ dysfunction syndrome was as high as 38.9%.

**Conclusion/significance:**

Scrub typhus, a common acute febrile illness in India causing severe morbidity, accounts for a large number of deaths. The burden of the disease has been underappreciated. Early diagnosis and prompt treatment can significantly reduce complications and mortality. Establishing good surveillance and instituting appropriate control measures are urgently needed.

## Author summary

Scrub typhus is an infection that is caused by a rickettsial bacteria called *Orientia tsutsugamushi* and transmitted to humans through chigger mites. Scrub typhus has re-emerged as one of the most common life-threatening rickettsial infections accounting for a million cases annually. However, it is a largely overlooked public health problem in India.

This systematic review aims to integrate data obtained from medical literature over the past 10 years to estimate the burden of scrub typhus in India. It also intends to summarize the diagnostic tests that are most commonly used to confirm scrub typhus cases and to map the distribution of cases across India. The insights provided from this review will further enable policymakers to establish a surveillance system to observe this disease and initiate strategies to effectively control, and prevent the spread of, this neglected disease.

In this review, we found that scrub typhus is very common in India and affects multiple organs in the majority of positively tested patients. This increases complications and the risk of death. Improving awareness about this disease, increasing access to testing and initiating appropriate therapy early can help reduce this public health threat in the future.

## Introduction

Scrub typhus, caused by the bacteria *Orientia tsutsugamushi* and transmitted by *Leptotrombidium* mites, is responsible for a potentially fatal tropical infection which is a grossly under-recognized public health problem in India[1,2]. About a million cases of scrub typhus are reported annually and the disease is associated with high mortality[3]. The disease is known to be endemic to the geographically confined area of the Asia-Pacific region termed as the ‘tsutsugamushi triangle’, which covers South and Southeast Asia, Northern Australia, and the islands of the Indian and Pacific Oceans[1]. However, there have been recent reports of scrub typhus from Chile, Peru, Africa, and the Arabian Peninsula, suggesting that there is a wider spread of infection[1,4].

There has been a resurgence of scrub typhus across India in the recent years; and scrub typhus has re-emerged as a major cause of acute undifferentiated febrile illnesses (AUFI) with high morbidity and mortality[5,6]. This disease is known to occur in diverse ecological settings in India with large numbers of cases being reported from Tamil Nadu, Andhra Pradesh, Karnataka, and Kerala in the South, Himachal Pradesh, Uttaranchal, Jammu, and Kashmir in the North, Meghalaya, Assam, and Nagaland in the North-East, West Bengal and Bihar in the East, and Maharashtra and Rajasthan in the West[7].

Scrub typhus commonly manifests with fever, breathlessness, cough, headache, nausea/vomiting, and altered sensorium. In some areas of the country, scrub typhus accounts for up to 35–50% of acute undifferentiated febrile illnesses requiring hospital admissions[8,9]. About a third of the scrub typhus cases requiring hospitalization have multi-organ dysfunction with pulmonary, hepatic, cardiac, neurological, or renal complications leading to high fatality rates[6,10]. Studies from India reveal that the case fatality rate (CFR) of scrub typhus ranges from 1.3% to 33.5% depending on the organ involvement and complications present[11,12,13].

Although scrub typhus is a public health threat in India, its national burden and distribution remain unclear due to the paucity of data and appropriate surveillance systems. However, estimating the burden of scrub typhus in India can lead to better control and management strategies. Hence, this systematic review aims to understand the magnitude and mortality of scrub typhus by incorporating data on the burden of scrub typhus in India available from literature over the past 10 years. It also intends to summarize the diagnostic tests that are most commonly used to confirm scrub typhus cases and to map the distribution of cases across India.

## Methods

This study was performed according to the PRISMA statement (**Appendix A in S1 File**: PRISMA Checklist)[14]. The protocol was registered in PROSPERO (CRD42020198079). The literature search strategies were developed using MeSH terms and keywords related to scrub typhus. An extensive literature search of two databases, PUBMED and SCOPUS were included to cover maximum number of articles published in International journals and Indian journals respectively. PUBMED and SCOPUS, restricted to the English language, human subjects, and India, and published over the last 10 years (2010–2020) was performed on 1^st^ September 2020. The search terms included ‘scrub typhus’, ‘*Orientia tsutsugamushi’*, or ‘Rickettsia’, used with ‘AND India’. A second search included ‘scrub typhus’ used with ‘AND’ and either ‘prevalence’, ‘incidence’, ‘epidemiology’, ‘mortality’, or ‘death’. Articles on all observational studies, cohort studies, case-control studies, case series, and case reports as well as interventional studies, including randomized controlled trials (RCT’s) and non-randomized clinical trials that reported or assessed laboratory-confirmed scrub typhus, conducted in any state in India, among patients of all age groups were included. Case reports and case series with less than five cases were excluded. Studies on diagnostic evaluation and epidemiological factors associated with scrub typhus were also included.

The primary outcomes evaluated were the number of cases diagnosed and the number of deaths due to scrub typhus. The diagnostic confirmation used in the study, such as IgM or IgG detection using ELISA, immunofluorescence test, rapid diagnostic test (RDT), Weil-Felix test, or polymerase chain reaction (PCR) were searched and documented.

### Case Definitions:

- Scrub typhus cases: “Patients with a febrile illness with or without an eschar confirmed by a molecular/serological diagnostic test”.
- Case fatality proportion: The proportion of deaths among individuals diagnosed with scrub typhus.
- Sero-epidemiology of scrub typhus: “Sero-epidemiology study of scrub typhus using IgG test”
- Organ Involvement:
  ■ Acute respiratory distress syndrome (ARDS) was defined as the patients with PaO2/FiO2 ratio<200 mmHg and/or low saturation with bilateral infiltrates on a chest radiograph in the absence of heart failure/cardiomegaly.
  ■ Hepatitis was defined as the total bilirubin >2mg/dl with elevation of AST (aspartate aminotransferase) /ALT (alanine aminotransferase).
  ■ AKI (Acute kidney injury) was defined as a serum creatinine level over 1.5 mg/dL.
  ■ Shock was defined as an arterial systolic blood pressure below 90 mmHg and/or refractory shock requiring inotropes.
  ■ Thrombocytopenia was defined as a platelet count below 100000/cmm
  ■ Myocarditis was defined as the elevated CKMB & Troponin T, abnormal electrocardiography
  ■ Meningitis was defined as neurological symptoms including headache, altered sensorium, seizure, and vomiting with or without CSF analysis indicating elevated protein level with no other evident causes.
  ■ Multi organ dysfunction syndrome (MODS) is defined as dysfunction of two or more organ systems.

### Outcome Measures:

- The overall number of confirmed scrub typhus cases in India
- The overall case fatality rate of scrub typhus in India
- The proportion of scrub typhus cases reported among patients with acute undifferentiated febrile illness
- The seroprevalence of scrub typhus in the community
- The regional distribution of scrub typhus reported in India

### Selection of the included studies and data extraction

Based on the eligibility criteria, two independent reviewers initially screened the titles and abstracts. Duplicates were removed using Zotero software. Subsequently, full articles were obtained. A study was included when both reviewers independently assessed the full text and found it satisfied the inclusion criteria. If there was any disagreement in selecting studies between the two reviewers, a third independent reviewer was consulted for the final decision.

Piloting was done before data extraction. Once the studies were identified for inclusion, the following data were extracted into a database: the study ID (author surname, year of publication), total number of participants or the sample size, place in which the study was conducted, source and setting of the population, regional distribution of cases, study design, whether the study was hospital or community based, diagnostic tests or criteria used, number of scrub typhus cases, and demographic data including age group, mean age, gender, occupation, and location (rural or urban). The number of fatal outcomes was also documented.

### Risk of bias assessment

The studies were assessed for methodological quality using a data extraction sheet designed for this review. The risk of bias for each included study was independently assessed by the same two initial reviewers. The third reviewer mediated in situations of disagreement. The risk of bias was ascertained using a quality assessment checklist for prevalence studies (adapted from Hoy et al.) based on which the studies were classified into low, moderate, and high-risk studies [15]. (Appendix B in S1 File: Risk of bias assessment Scale)

### Strategy for data synthesis

A systematic narrative synthesis was conducted to summarize the included studies in the text and tables.

## Results

**Fig 1:**
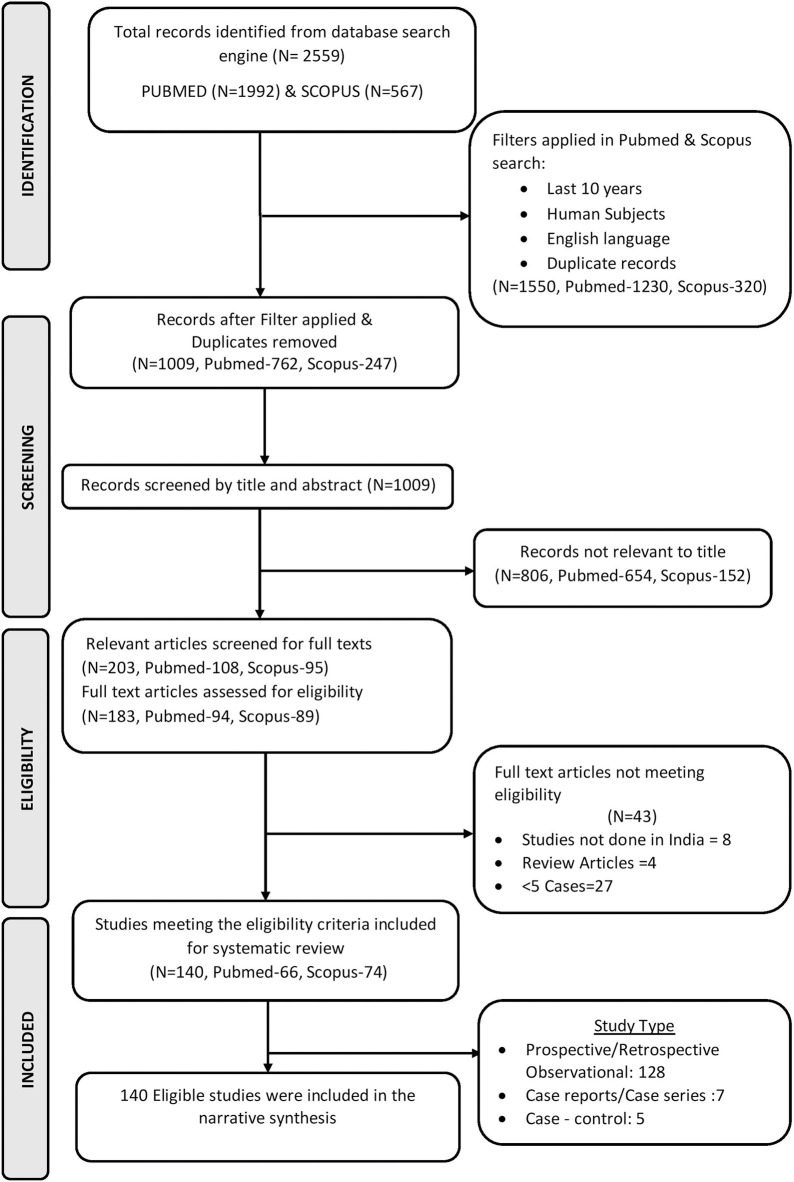
PRISMA Flowchart (Schematic representation of the study). This figure describes the details of the data extraction process which describes and summarises the total number of articles identified from the search engines (PUBMED (N) - 1992, SCOPUS (N) - 567); total articles screened after filters applied and duplicates removed (N—1009); total number of full text articles assessed for eligibility (N—183) and the total number of articles included in the narrative synthesis (N—140).

The search strategy initially identified 2559 articles from the two databases out of which 183 full-text articles met the eligibility criteria and were identified for inclusion. After the review of full-text articles, 140 articles were found to report laboratory-confirmed scrub typhus cases which included 138 studies conducted in a hospital setting and two seroprevalence studies. A total of 89 articles evaluated undifferentiated febrile illness including scrub typhus. The details of the eligible studies are included in the PRISMA flowchart (Fig 1). A total of 81 studies had low risk and 52 studies had a moderate risk of bias with the relevant data. One study had a high risk of bias with some missing data.

### Diagnostic methods used for the confirmation of scrub typhus cases

Studies using a serological or molecular method for the confirmation of scrub typhus cases were included in the analysis. IgM ELISA was used in 122 studies and confirmed the majority (89%) of scrub typhus cases. PCR was used to confirm 17.9% of cases. Other confirmatory diagnostic tests used were Weil Félix (20%), IFA (5.5%), and RDT (1.4%). IgG ELISA was used in four studies to confirm 1.1% of scrub typhus cases, and ten studies had also used the presence of an eschar for diagnosing scrub typhus cases along with other laboratory diagnostic methods. More than one diagnostic method for case confirmation was used in 58 studies. The usage of the Weil Felix test as a confirmatory diagnostic aid saw a steady decline from 2014 onwards.

### Estimates of the burden of scrub typhus

During the last 10 years, there were 18,781 confirmed scrub typhus cases published in 140 medical literatures out of a total of 1,23,067 participants with acute febrile illness from 138 hospital-based studies and 2 community-based studies. Among the 89 studies comprising of participants with AUFI, 10,885 had cases of lab-confirmed scrub typhus causing an estimated proportion of 25.3% among individuals with AUFI. The two community-based studies estimated seroprevalences of 40.3% and 31.8%, respectively, with an average of 34.2% among healthy individuals. The summary of articles included, and the cases of scrub typhus, are provided in Table 1.

**Table 1:** Overall Burden of Scrub typhus in India.

Overall Burden	No of studies	No of scrub patients/ Total no of participants (%)
**All studies**	140	18781/123067 (15.3%)
**Hospital based studies**	138	18431/122046 (15.1%)
**AUFI studies**	89	10885/43106 (25.3%)
**Seroprevalence studies**	2	350/1021 (34.2%)

### Epidemiology and regional distribution of scrub typhus cases

The age distribution of laboratory-confirmed scrub typhus cases was reported in 133 studies of which 43 were done in all age groups. The pooled median age of participants with laboratory-confirmed scrub typhus was found to be 28.1 years. Among all the scrub typhus cases reported in 140 studies, 20.3% of them were reported to be at or below 15 years of age, 47.1% of the cases were reported to be above 15 years of age and 32% of the cases included all age groups or did not specify the age groups.

Data on gender was available in 107 studies (74.5%), and cases were equally distributed between males (50.1%) and females (49.9%). The occupations of laboratory-confirmed cases were reported in 32 studies, and 53.3% of cases were agricultural laborers or unskilled workers. Data on the place of stay was available in 34 studies. A vast majority of cases (81.7%) resided in rural areas close to shrubs and bushes as compared with the 18.3% who resided in urban settlements. The median values across patient series and their respective ranges are presented in Table 2.

**Table 2:** Demographics and clinical characteristics across patient series.

Variables	Number of studies	Number of patients / Total no of cases	Median value across patient series (Range)
**Age** (in years) (Range)	133	16982/18781(90.4%)	**28.1 years** (0.1–105)
≤15 years	33	3448/16982(20.3%)	-
> 15 years	57	7997/16982(47.09%)	-
All age groups	43	5537/16982(32.6%)	-
**Gender**	107	13991/18781(74.4%)	
Males	107	7007/13991(50.1%)	52.9%(15.5–100)
**Occupation**	32	3846/18781(20.5%)	
Agricultural labourer	28	2041/3846(53.3%)	42.2%(11.1–89.4)
Housewife/unemployed	16	933/3846(24.3%)	41.4%(14.9–94.2)
Self-employed/Students/ Others	19	864/3846(22.4%)	26.7%(7.9–57.8)
**Place of residence**	34	3318/18781(17.7%)	100%(72.5–100)
Rural	32	2710/3318(81.7%)	94.0%(18.6–100)
Urban	17	608/3318(18.3%)	39.1%(3.3–100)
**Clinical characteristics**	93	12675/18781(67.5%)	
Fever	65	6518/18781(34.7%)	100%(13.3–100)
Mean duration of Fever	34	2791/6518(42.8%)	8.8 days(4–14 days)
<7 days	24	862/2206(39.07%)	-
7–14 days	26	1726/2803(61.6%)	-
>14 days	15	204/1479(13.8%)	-
Eschar	94	3707/18781(19.7%)	22.1%(1–100)
Rash	53	934/18781(5%)	14.3%(1.32–100)
Headache	68	3421/18781(18.2%)	39%(1.5–94.9)
Nausea/Vomiting	68	3214/18781(17.1%)	38.9%(1.2–100)
Altered Sensorium	55	1378/18781(7.3%)	19.5%(0.5–83.8)
Seizure	34	514/18781(2.7%)	6.3%(0.4–47.9)
Abdominal pain	63	1972/18781(10.5%)	27.3%(4.3–100)
Cough	48	1944/18781(10.4%)	30.2%(1.7–84.6)
Breathlessness	46	1961/18781(10.4%)	26.2%(2.1–100)
Jaundice/ Icterus	34	1049/18781(5.6%)	14%(1.8–83.3)
**Diagnostic confirmation**	140	18781/18781(100%)	
IgM ELISA	122	16780/18781(89.3%)	48.8%(0.6–100)
IFA	9	1041/18781(5.5%)	30.8% (10.2–100)
IgG ELISA	4	198/18781(1.1%)	36% (15–100)
WF	46	3759/18781(20%)	30.9%(4–100)
ICT	4	258/18781(1.4%)	100% (30.9–100)
PCR	23	3361/18781(17.9%)	38.9%(0.6–100)

IgM & IgG ELISA—Enzyme-Linked Immunosorbent Assay; IFA–Immunofluorescence assay; WF–Weil-Felix; ICT- Immunochromatographic test; PCR- Polymerase chain reaction

**Fig 2:**
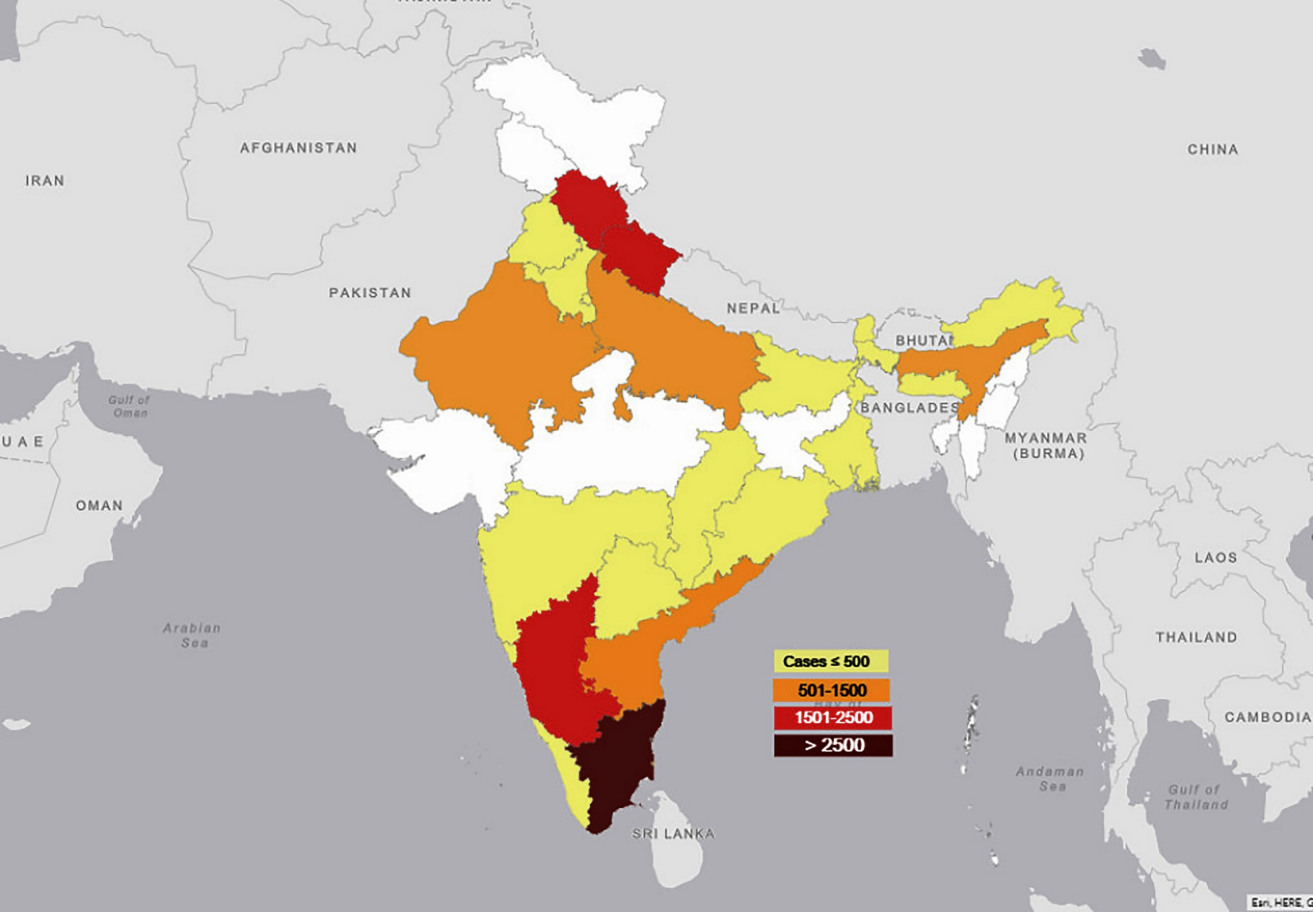
Mapping the regional distribution of scrub typhus in India. Number of scrub typhus cases less than or equal to 500 is represented as yellow, 501–1500 cases is represented as orange, 1501–2500 cases is represented as red and more than 2500 cases is represented as maroon. This map was created in the free version of ARC GIS by the first author. Please see the ARC GIS link https://arcg.is/1iDvKu. The base layer map was used from the Survey of India, Department of Science & Technology which gives open access to the general public https://indiamaps.gov.in/soiapp/.

There were a total of 64 studies from South India, 46 studies from North India, 13 from North-East India, 13 from the East, and four from the West reporting 55.5%, 31.5%, 7.4%, 4.5%, and 1.1% of scrub typhus cases, respectively. The state-wise proportion of cases from each region are summarised in Table 3 and Fig 2.

**Table 3:** Regional distribution of Scrub typhus in India.

Region wise burden of scrub typhus in India	No. of studies	No of scrub patients / Total no of study participants	Proportion of cases (%)
**South**	**64**	**10434/94961 (11%)**	**10434/18781 (55.5%)**
Tamilnadu	34	7067/85301 (8.3%)	7067/ 18781 (37.6%)
Puducherry	10	809/1707 (47.4%)	809/18781 (4.3%)
Andhra Pradesh	5	596/1335 (44.6%)	596/18781 (3.2%)
Telengana	2	185/775 (24%)	185/18781 (1%)
Karnataka	12	1669/3559 (46.9%)	1669/18781 (8.8%)
Kerala	1	108/2682 (4%)	108/18781 (0.6%)
**North**	**46**	**5919/19604 (30.2%)**	**5919/18781 (31.5%)**
Chandigarh	6	285/2012 (14.2%)	285/18781 (1.5%)
Delhi	3	78/295 (26.4%)	78/18781 (0.4%)
Haryana	1	39/230 (17%)	39/18781 (0.2%)
Himachal Pradesh	11	2072/2156 (96.1%)	2072/18781 (11%)
Punjab	2	160/834 (19.2%)	160/18781 (0.9%)
Rajasthan	6	556/1046 (53.2%)	556/18781 (3%)
Uttrakhand	10	1593/9585 (16.6%)	1593/18781 (8.5%)
Uttar Pradesh	7	1136/3446 (33%)	1136/18781 (6%)
**East**	**13**	**835/3082 (27.1%)**	**835/18781 (4.5%)**
Bihar	1	140/763 (18.3%)	140/18781 (0.8%)
Chattisgarh	1	23/223 (10.3%)	23/18781 (0.1%)
Odisha	4	406/981 (41.4%)	406/18781 (2.2%)
West Bengal	7	266/1115 (23.9%)	266/18781 (1.4%)
**North east**	**13**	**1384/4280 (32.3%)**	**1384/18781 (7.4%)**
Assam	5	778/2775 (28%)	778/18781 (4.1%)
Arunachal Pradesh	1	121/300 (40.3%)	121/18781 (0.7%)
Meghalaya	5	417/996 (42%)	417/18781 (2.2%)
Sikkim	2	68/209 (32.5%)	68/18781 (0.4%)
**West**	**4**	**209/667 (31.3%)**	**209/18781 (1.1%)**
Goa	2	36/99 (36.4%)	36/18781 (0.2%)
Maharashtra	2	173/568 (30.5%)	173/18781 (0.9)

Thirty-two studies reported the month of testing. Seasonally, the highest number of cases was observed during the cooler months of the year, between August and January.

### Clinical characteristics of laboratory-confirmed scrub typhus cases

A total of 93 studies had data on the clinical characteristics of scrub typhus. The most common presenting symptoms of confirmed cases were headache (18.2%) followed by nausea/vomiting (17.1%), abdominal pain (10.5%), breathlessness (10.4%), cough (10.4%), jaundice (5.6%), and seizure (2.7%). Data on the presence of fever was reported in 65 studies and the duration of fever was stated in 34 studies and showed a median duration of 8.8 days (4–14 days). Fever duration of less than 7 days was reported in 24 studies in 39.1% of the participants. Fever duration of more than 14 days at admission was reported in 15 studies comprising 13.8% of the participants. The presence of a pathognomic eschar was reported in 94 studies and was noted among 22% of cases. Median percentages and range values of the clinical characteristics are summarized in Table 2.

### Organ involvement and case fatality rates

Severe scrub typhus cases with various organ involvement were reported in 91 studies with a total of 11,535 scrub typhus cases. The most common complications included hepatitis (40.5% of cases of scrub typhus), thrombocytopenia (28.4%), acute respiratory distress syndrome or ARDS (20.5%), acute kidney injury (19.2%), meningitis (16.4%), shock (16.2%), and myocarditis (15.5%). The number of studies reporting each complication is compiled in Table 4. Multiple organ dysfunction syndromes (MODS) were reported in 33 studies and were seen in 17.4% of the cases. As reported by 18 and 20 studies, respectively, 20.4% patients required ICU admission and 19.1% required ventilation.

**Table 4:** Organ involvement & mortality.

Complications Summary	No. of studies	No of patients / Total cases	Proportion % (Range)	Case Fatality Rate(CFR) No of deaths / Total no of cases
**Organ involvement**	91	-	-	6.2% (551/8846; 63 studies)
ARDS	61	1671/8148	20.5% (0.6–96.5)	26.8%(445/1655; 52 studies)
AKI	53	1196/6222	19.2% (1.2–80)	34.6%(348/1005; 41 studies)
Hepatitis	45	1701/4197	40.5% (3.3–86.9)	23.2%(242/1043; 20 studies)
Shock	40	884/5454	16.2% (2.0–44.8)	39.6%(130/328; 18 studies)
Myocarditis	22	487/3152	15.5% (0.4–56.9)	42.4%(189/446;16 studies)
Meningitis	54	1217/7419	16.4% (1.7–100)	35.5%(250/705; 25 studies)
Thrombocytopenia	38	1074/3786	28.4% (0.3–97.7)	21.9%(167/763; 23 studies)
MODS	33	851/4903	17.4% (0.4–100)	38.9%(231/ 593; 28 studies)
Patients requiring ICU admission	18	680/3335	20.4%(5–100)	29.7%(62/209); 2 studies
Patients requiring ventilation	20	471/2469	19.1%(2–100)	19.2%(10/52); 1 study
**Case Fatality Rate**				
Overall	75	-	-	6.3% (658/10478)
AUFI studies	40	-	-	7.6% (403/5279)

ARDS—Acute respiratory distress syndrome; AKI—Acute kidney injury: MODS—Multi organ dysfunction; ICU—Intensive care unit; AUFI- Acute undifferentiated febrile illness

The overall case fatality rate from 75 studies was found to be 6.3% and from the 40 AUFI studies was 7.6%. Regionally, the highest CFR was 8.5%, reported in North India, followed by 4.7%, reported in South India. However, the proportion of scrub typhus cases was higher in South India (55.5%) than in North India (31.5%). High case fatality rates were reported in patients with myocarditis (42.4%) followed by shock (39.6%), meningitis (35.5%), acute kidney injury (34.6%), ARDS (26.8%), hepatitis (23.2%), and thrombocytopenia (21.9%). Information on death among MODS cases was noted in 28 studies and was found to be 38.9%. Data on CFR in individual organ involvement is summarized in Table 4.

## Discussion

**Fig 3:**
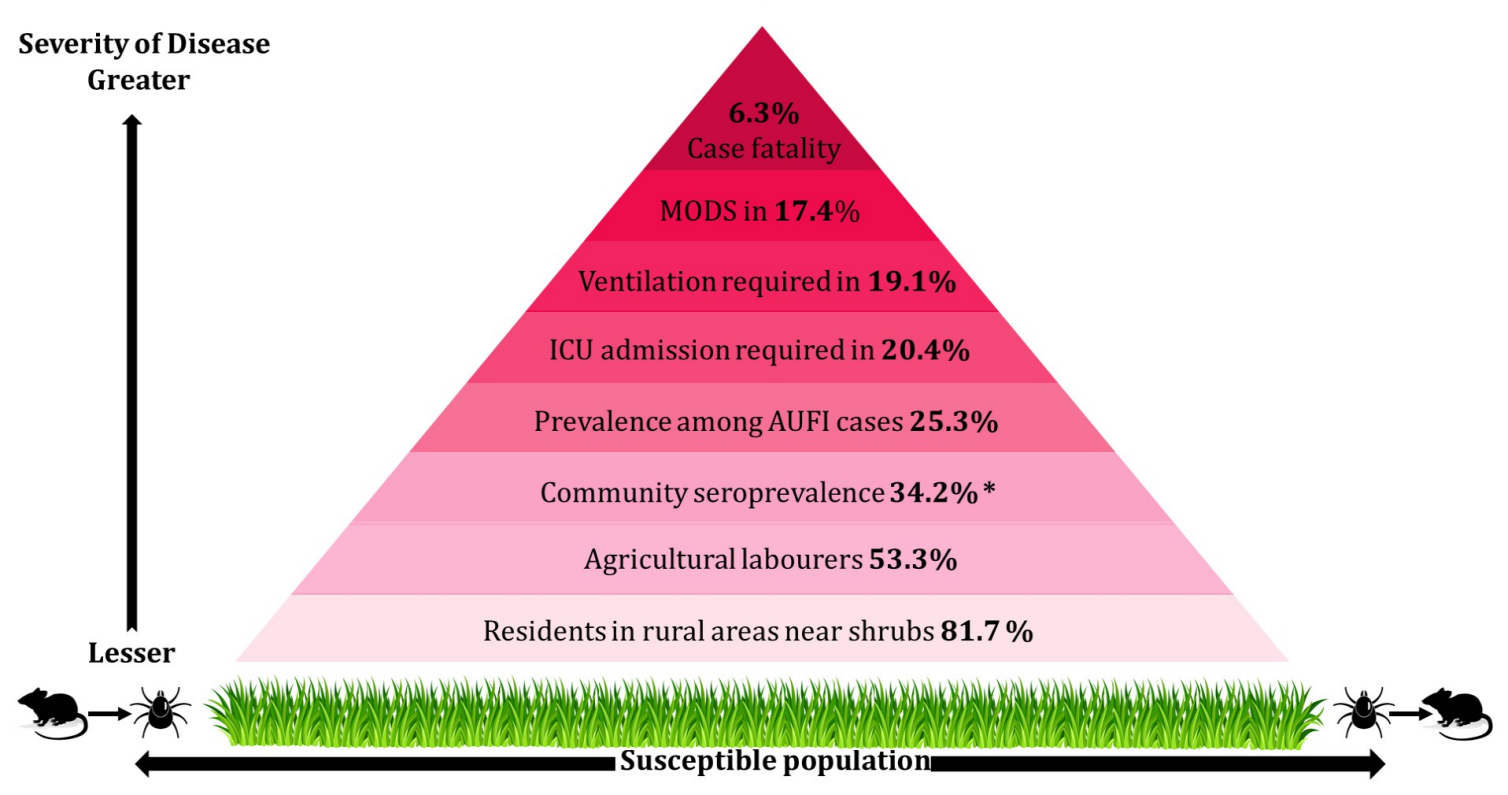
Summary of significant findings relating to the Burden of Scrub typhus in India. Scrub typhus is vector borne febrile illness in susceptible population caused by chigger bites harbouring *Orientia tsutsugamushi*. It was noted in this study that 81.7% of residents from rural areas exposed to shrubs are affected. Agricultural labourers (53.3%) were at the highest risks of contracting the illness. Community sero prevalence was reported to be 34.2% in India (*this was based on 2 community based study). 25.3% of all the hospitalised AUFI are reported to be scrub typhus. Among the severe scrub typhus cases 20.4% needed ICU admission and 19.1% were eventually put on ventilation. Multi organ dysfunction syndrome was reported in 17.4% of the patients. Scrub typhus has a case fatality rate of 6.3%. Further epidemiological studies need to be carried out to suffice the dearth of evidence of scrub typhus seroprevalence and severity in the community. The figure was created in Microsoft Paint and PowerPoint. The glass blades and mice silhouette are taken from https://openclipart.org/detail/164635/grass and https://www.publicdomainpictures.net/en/view-image.php?image=145546&picture=mouse-silhouette-sitting respectively and modified in Paint to get desirable effect.

With growing numbers of cases detected in India, scrub typhus is fast emerging as a public health threat. However, its burden remains elusive as it is vastly underdiagnosed and underreported. This systematic review collated published literature from the past decade on the burden of scrub typhus, its regional differences, and mortality to help guide and inform national and global health policies. The vast majority of published literature included in the analysis was hospital-based studies (138 out of 140 studies). Eighty-nine studies evaluated acute undifferentiated febrile illness and documented scrub typhus in 25.3%. The case fatality rate from various studies was estimated to be around 7% overall, but increased significantly to 40% with organ involvement, such as myocarditis, shock, and multiorgan dysfunction syndrome. The significant findings of the study have been represented pictorially in Fig 3.

Scrub typhus is reported as the leading cause of treatable non-malarial febrile illness by prospective studies of acute febrile illness from Asia[1,16]. The mean estimate of scrub typhus (25.3%) among the studies reporting AUFI in this review is similar to the burden of scrub typhus among the patients presenting to hospitals with fever that is seen in large studies from India[2,9]. Though the number of cases reported varied across the years, this review showed that there was an increasing trend of scrub typhus over time in India, suggesting a worsening situation since 2010. However, the increase in the use of better confirmatory diagnostic tests especially scrub typhus ELISA and RT-PCR beyond 2014 has helped in validating the scrub typhus cases better which could be a contributory factor for the increasing numbers. The available literature on sero-epidemiological data suggests that *Orientia tsutsugamushi* infection is common across Asia, with a median seroprevalence of 22.2%[1]. The two community-based studies conducted during the period in this review (2016 and 2017, respectively) showed seroprevalences of 40.3% and 31.8%[17,18]. A recent study among urban areas, rural plains, and peri-forest hill villages in Tamil Nadu showed a seroprevalence of 28.1%[19]. The data so far suggests that the seroprevalence of scrub typhus in the community is about 30% which is comparable to endemic infections like tuberculosis in India, which has an estimated prevalence (latent tuberculosis) of 40%[20]. Thus, it is likely that the actual cases of scrub typhus are substantially higher than what is diagnosed and reported, and what is summarized in this study likely represents only the tip of the iceberg.

The epidemiology of scrub typhus is variable geographically based on the different climatic conditions and the species diversity of etiological agents as well as arthropod vectors involved in transmission[21,22]. In this review, the epidemiology of scrub typhus within the country and the regional distribution were heterogeneous with the maximum proportion of cases being reported from South India (55.5%) followed by North India (31.5%). This may be partially attributable to awareness about the disease and the availability of diagnostic facilities in South India. A huge majority (81.7%) of scrub typhus cases was reported from rural areas in India, which is higher than the proportion of cases from rural areas (65.0%) reported from some of the countries in Southeast Asia[7]. The monsoon and post-monsoon seasons between July and February corresponded with high occurrences of scrub typhus cases in most states in India as reported in 32 studies.

The findings of the study indicate that most of the laboratory-confirmed cases of scrub typhus in India occur in young adults exposed to scrub vegetation with a median age of 28.1 years. IgM ELISA, an accurate and accessible means of diagnosis, was used as the confirmatory test in a large proportion of studies (122 studies) and detected 87% of all cases. In a recent study evaluating the performance of molecular and serologic tests for the diagnosis of scrub typhus, ELISA was found to have a sensitivity of 94.2% and a specificity of 93.6%, which makes it a reliable test[23]. Further, when compared with PCR, IgM ELISA is less expensive. In our analysis, 17.9% of cases were confirmed by PCR, and about 20% of the cases were confirmed by the Weil-Felix test, which is known to have poor sensitivity. Beyond 2014, ELISA and PCR were the most commonly used confirmatory tests either individually or in combination with other confirmatory tests and eschar. From 2010–2014, 28 studies used Weil-Felix in diagnosing scrub typhus but from 2014–2020 Weil-Felix was used only in 18 studies as a confirmatory diagnostic test. Therefore, the use of the Weil-Felix test appears to have reduced drastically after 2014.

Case fatality rates of scrub typhus reported in literature varied widely around a median of 6.0%[1,3,24]. In this analysis, the case fatality rates from 75 studies that had data on deaths were similar and the overall CFR was 6.3%. The CFR of the 40 AUFI studies was 7.6%. The case fatality can be as high as 30% or more depending on the presence of complications and administration of appropriate treatment[7,21]. In this analysis, multiple organ dysfunction syndrome was reported in 33 studies and was seen in 17.4% of all cases. This was slightly higher than that in another systematic review looking at the clinical manifestations of scrub typhus in South East Asia which reported MODS in 15% of cases[24]. In severe cases with multi-organ failure, mortality is reported to be around 24% despite therapy[25]. Case fatality rates varied between regions and states. Regions with well-established health systems with easy accessibility showed lower mortality rates as compared to states with limited facilities. This review revealed that while the maximum number of cases was reported from South India, the maximum number of deaths was reported in North India. The lower mortality rates in South India may be attributed to the presence of well-established health systems as compared to the limited facilities present in the northern states. Additionally, the higher case fatality rates in the northern region could also be attributed to delay in seeking medical care.

The findings of this study confirm that scrub typhus is a life-threatening disease which is geographically expanding over time. This calls for a well-established surveillance system along with collaborative actions in endemic regions using early diagnostic strategies to effectively control, and prevent the spread and outbreaks of, this neglected disease. Further, this study showed that case fatality rates are higher with complications that can be prevented through early diagnosis and prompt treatment. Improving awareness of the disease, point-of-care diagnosis, and appropriate therapy can help reduce this public health threat in the future.

### Limitations

This study included an extensive search and amalgamation of all relevant literature on scrub typhus in the last decade. However, it used only two search engines, which contains most of the published literature on studies done in India: PUBMED and SCOPUS. The study showed that there was significant variation in the burden of scrub typhus within the prospective fever studies (median, 35.4%; ranging from 4% - 96.8%) which reflects a lack of uniformity due to different diagnostic modalities and patient selection methods used. This is similar to the findings of Ana Bonell et al., estimating the global burden of scrub typhus, which found a median proportion of 23.4% with a wide range of 1–96.9% among fever studies[1]. This review included mainly hospital-based studies and only 2 community-based studies. Further studies including wider seroprevalence in the community and well planned prospective evaluation among patients who present with AUFI across the country are needed to understand precise burden and institute appropriate control and prevention measures.

## Conclusion

The overall proportion of scrub typhus among patients with AUFI were 25.3%. The average community seroprevalence was 34.2%. The highest densities of cases were from Tamil Nadu (37.6% of cases), Himachal Pradesh (11%), Karnataka (8.8%), and Uttrakhand (8.5%). Scrub typhus affected males and females equally but was more prevalent in agricultural laborers/unskilled workers (53.3%) and those residing in rural areas (81.7%). The most commonly noted among multiple organ involvement was hepatitis (40.5%). MODS were reported in 17.4%; 20.4% patients required ICU admission; and 19.1% required ventilation. The overall case fatality rate was 6.3% and increased to 7.6% among patients with AUFI. CFR was high in patients with myocarditis (42.4%), shock (39.6%), MODS (38.9%), meningitis (35.5%), acute kidney injury (34.6%), ARDS (26.8%), and hepatitis (23.2%). With regards to the diagnostic tests, IgM ELISA was most commonly used (89%). These findings suggest that there is a high burden of scrub typhus and that early diagnosis and prompt treatment can significantly reduce the complications and case fatality of scrub typhus.

## Supporting information

S1 File Supporting Information. **Appendix A in S1 File—PRISMA—P 2015 Checklist [14].** This checklist has been adapted for use with protocol submissions to *Systematic Reviews* from Table 3 in Moher D et al: Preferred reporting items for systematic review and meta-analysis protocols (PRISMA-P) 2015 statement. *Systematic Reviews* 2015 4:1**. Appendix B in S1 File—Risk of bias assessment Scale [15].** Quality assessment checklist for prevalence studies (adapted from Hoy et al).
(DOCX)

## References

[1] Bonell A, Lubell Y, Newton PN, Crump JA, Paris DH. Estimating the burden of scrub typhus: A systematicreview. *PLoS Negl Trop Dis*. 2017 Sep 25;11(9):e0005838. doi: 10.1371/journal.pntd.0005838 ; PMCID: PMC5634655. 28945755 PMC5634655

[2] Behera B, Biswal M, Das RR, Dey A, Jena J, Dhal S, et al. Clinico-epidemiological analysis of scrub typhus in hospitalised patients presenting with acute undifferentiated febrile illness: A hospital-based study from Eastern India. *Indian J Med Microbiol*. 2019 Apr-Jun; 37(2):278–280. doi: 10.4103/ijmm.IJMM_19_147 . 31745031

[3] Taylor AJ, Paris DH, Newton PN. A Systematic Review of Mortality from Untreated Scrub Typhus (Orientia tsutsugamushi). PLoS Negl Trop Dis. 2015 Aug 14;9(8):e0003971. doi: 10.1371/journal.pntd.0003971 ; PMCID: PMC4537241. 26274584 PMC4537241

[4] Walker DH. Scrub Typhus—Scientific Neglect, Ever-Widening Impact. N Engl J Med. 2016 Sep 8;375(10):913–5. doi: 10.1056/NEJMp1608499 . 27602663

[5] Kispotta R, Kasinathan A, Kumar Kommu PP, Manikandan M. Analysis of 262 Children with Scrub Typhus Infection: A Single-Center Experience. Am J Trop Med Hyg. 2020 Nov 9. doi: 10.4269/ajtmh.20-1019 Epub ahead of print. . 33219642 PMC7866334

[6] Varghese GM, Trowbridge P, Janardhanan J, Thomas K, Peter JV, Mathews P, Abrahamet al. Clinical profile and improving mortality trend of scrub typhus in South India. Int J Infect Dis. 2014 Jun; 23:39–43. doi: 10.1016/j.ijid.2014.02.009 Epub 2014 Mar 21. . 24661931

[7] Xu G, Walker DH, Jupiter D, Melby PC, Arcari CM. A review of the global epidemiology of scrub typhus. PLoS Negl Trop Dis. 2017 Nov 3;11(11):e0006062. doi: 10.1371/journal.pntd.0006062 ; PMCID: PMC5687757. 29099844 PMC5687757

[8] Bal M, Mohanta MP, Sahu S, Dwibedi B, Pati S, Ranjit M. Profile of Pediatric Scrub Typhus in Odisha, India. Indian Pediatr. 2019 Apr 15;56(4):304–306. . 31064899

[9] Abhilash KP, Jeevan JA, Mitra S, Paul N, Murugan TP, Rangaraj A, et al. Acute Undifferentiated Febrile Illness in Patients Presenting to a Tertiary Care Hospital in South India: Clinical Spectrum and Outcome. J Glob Infect Dis. 2016 Oct-Dec;8(4):147–154. doi: 10.4103/0974-777X.192966 ; PMCID: PMC5126753. 27942194 PMC5126753

[10] Sharma N, Biswal M, Kumar A, Zaman K, Jain S, Bhalla A. Scrub Typhus in a Tertiary Care Hospital in North India. Am J Trop Med Hyg. 2016 Aug 3;95(2):447–51. doi: 10.4269/ajtmh.16-0086 Epub 2016 Jun 13. ; PMCID: PMC4973198. 27296391 PMC4973198

[11] Narvencar KPS, Rodrigues S, Nevrekar RP, Dias L, Dias A, Vaz M, et al. Scrub typhus in patients reporting with acute febrile illness at a tertiary health care institution in Goa. Indian J Med Res. 2012 Dec;136(6):1020–4. 23391799 PMC3612306

[12] Takhar RP, Bunkar ML, Arya S, Mirdha N, Mohd A. Scrub typhus: A prospective, observational study during an outbreak in Rajasthan, India. Natl Med J India. 2017 Apr;30(2):69–72. 28816212

[13] Sivaprakasam E, Rajan M, Pasupathy U, Ravichandran L. Clinical Characteristics and Predictors of Severity of Pediatric Scrub Typhus in a Tertiary Level Hospital in South India. Archives of Pediatric Infectious Diseases. 2020; 8: doi: 10.5812/pedinfect.92752

[14] Moher D, Shamseer L, Clarke M, Liberati A, Petticrew M, et al. Preferred reporting items for systematic review and meta-analysis protocols (PRISMA-P) 2015 statement. *Syst Rev* 4, 1 (2015). doi: 10.1186/2046-4053-4-1 25554246 PMC4320440

[15] Hoy D, Brooks P, Woolf A, Blyth F, March L, Bain C, Baker P, Smith E, Buchbinder R. Assessing risk of bias in prevalence studies: modification of an existing tool and evidence of interrater agreement. J Clin Epidemiol. 2012 Sep;65(9):934–9. doi: 10.1016/j.jclinepi.2011.11.014 Epub 2012 Jun 27. . 22742910

[16] Thapa S, Hamal P, Chaudhary NK, Sapkota LB, Singh JP. Burden of scrub typhus among patients with acute febrile illness attending tertiary care hospital in Chitwan, Nepal. BMJ Open. 2020 Sep 17;10(9):e034727. doi: 10.1136/bmjopen-2019-034727 ; PMCID: PMC7500310. 32948542 PMC7500310

[17] Jakharia A, Borkakoty B, Biswas D, Yadav K, Mahanta J. Seroprevalence of Scrub Typhus Infection in Arunachal Pradesh, India. Vector Borne Zoonotic Dis. 2016 Oct;16(10):659–63. doi: 10.1089/vbz.2016.1970 Epub 2016 Aug 18. . 27536803

[18] Trowbridge P, P D, Premkumar PS, Varghese GM. Prevalence and risk factors for scrub typhus in South India. Trop Med Int Health. 2017 May;22(5):576–582. doi: 10.1111/tmi.12853 Epub 2017 Mar 2. . 28173608

[19] Devamani CS, Schmidt WP, Ariyoshi K, Anitha A, Kalaimani S, Prakash JAJ. Risk Factors for Scrub Typhus, Murine Typhus, and Spotted Fever Seropositivity in Urban Areas, Rural Plains, and Peri-Forest Hill Villages in South India: A Cross-Sectional Study. Am J Trop Med Hyg. 2020 Jul;103(1):238–248. doi: 10.4269/ajtmh.19-0642 Epub 2020 May 21. ; PMCID: PMC7356468. 32458785 PMC7356468

[20] Mandalakas AM, Kirchner HL, Lombard C, Walzl G, Grewal HM, Gie RP, et al. Well-quantified tuberculosis exposure is a reliable surrogate measure of tuberculosis infection. Int J Tuberc Lung Dis. 2012 Aug;16(8):1033–9. doi: 10.5588/ijtld.12.0027 Epub 2012 Jun 11. . 22692027 PMC11967560

[21] Rajapakse S, Rodrigo C, Fernando D. Scrub typhus: pathophysiology, clinical manifestations and prognosis. Asian Pac J Trop Med. 2012 Apr;5(4):261–4. doi: 10.1016/S1995-7645(12)60036-4 . 22449515

[22] Varghese GM, Janardhanan J, Mahajan SK, Tariang D, Trowbridge P, Prakash JA, et al. Molecular epidemiology and genetic diversity of Orientia tsutsugamushi from patients with scrub typhus in 3 regions of India. Emerg Infect Dis. 2015 Jan;21(1):64–9. doi: 10.3201/eid2101.140580 ; PMCID: PMC4285260. 25530231 PMC4285260

[23] Kannan K, John R, Kundu D, Dayanand D, Abhilash KPP, Mathuram AJ, et al. Performance of molecular and serologic tests for the diagnosis of scrub typhus. PLoS Negl Trop Dis. 2020 Nov 12;14(11):e0008747. doi: 10.1371/journal.pntd.0008747 ; PMCID: PMC7660479. 33180784 PMC7660479

[24] Rajapakse S, Weeratunga P, Sivayoganathan S, Fernando SD. Clinical manifestations of scrub typhus. Trans R Soc Trop Med Hyg. 2017 Feb 1;111(2):43–54. doi: 10.1093/trstmh/trx017 . 28449088

[25] Griffith M, Peter JV, Karthik G, Ramakrishna K, Prakash JA, Kalki RC, et al. Profile of organ dysfunction and predictors of mortality in severe scrub typhus infection requiring intensive care admission. Indian J Crit Care Med. 2014 Aug;18(8):497–502. doi: 10.4103/0972-5229.138145 ; PMCID: PMC4134622. 25136187 PMC4134622

