# Supplementary material for: The burden of scrub typhus in India: A systematic review
Source: PLoS Negl Trop Dis. 2021 Jul 27;15(7):e0009619. doi: 10.1371/journal.pntd.0009619 (PMC8345853; doi:10.1371/journal.pntd.0009619)
Supplement: S1 File — Appendix A in S1 File—PRISMA—P 2015 Checklist [14]. This checklist has been adapted for use with protocol submissions to Systematic Reviews from Table 3 in Moher D et al: Preferred reporting items for systematic review and meta-analysis protocols (PRISMA-P) 2015 statement. Systematic Reviews 2015 4:1. Appendix B in S1 File—Risk of bias assessment Scale [15]. Quality assessment checklist for prevalence studies (adapted from Hoy et al). (DOCX) [file pntd.0009619.s001.docx]

**Supporting Information**

**Appendix A in S1 File – PRISMA Checklist [1]**

| **Section/topic** | **#** | **Checklist item** | **Information reported** | | **Line number(s)** |
| --- | --- | --- | --- | --- | --- |
|  |  |  | **Yes** | **No** |  |
| **ADMINISTRATIVE INFORMATION** | | | | | |
| **Title** | | | | | |
| Identification | 1a | Identify the report as a protocol of a systematic review | ✓ |  |  |
| Update | 1b | If the protocol is for an update of a previous systematic review, identify as such |  | ✓ |  |
| **Registration** | 2 | If registered, provide the name of the registry (e.g., PROSPERO) and registration number in the Abstract | ✓ |  |  |
| **Authors** | | | | | |
| Contact | 3a | Provide name, institutional affiliation, and e-mail address of all protocol authors; provide physical mailing address of corresponding author | ✓ |  |  |
| Contributions | 3b | Describe contributions of protocol authors and identify the guarantor of the review | ✓ |  |  |
| **Amendments** | 4 | If the protocol represents an amendment of a previously completed or published protocol, identify as such and list changes; otherwise, state plan for documenting important protocol amendments |  | ✓ |  |
| **Support** | | | | | |
| Sources | 5a | Indicate sources of financial or other support for the review | ✓ |  |  |
| Sponsor | 5b | Provide name for the review funder and/or sponsor | ✓ |  |  |
| Role of sponsor/funder | 5c | Describe roles of funder(s), sponsor(s), and/or institution(s), if any, in developing the protocol | ✓ |  |  |
| **INTRODUCTION** | | | | | |
| **Rationale** | 6 | Describe the rationale for the review in the context of what is already known | ✓ |  |  |
| **Objectives** | 7 | Provide an explicit statement of the question(s) the review will address with reference to participants, interventions, comparators, and outcomes (PICO) | ✓ |  |  |
| **METHODS** | | | | | |
| **Eligibility criteria** | 8 | Specify the study characteristics (e.g., PICO, study design, setting, time frame) and report characteristics (e.g., years considered, language, publication status) to be used as criteria for eligibility for the review | ✓ |  |  |
| **Information sources** | 9 | Describe all intended information sources (e.g., electronic databases, contact with study authors, trial registers, or other grey literature sources) with planned dates of coverage | ✓ |  |  |
| **Search strategy** | 10 | Present draft of search strategy to be used for at least one electronic database, including planned limits, such that it could be repeated | ✓ |  |  |
| ***STUDY RECORDS*** | | | | | |
| Data management | 11a | Describe the mechanism(s) that will be used to manage records and data throughout the review | ✓ |  |  |
| Selection process | 11b | State the process that will be used for selecting studies (e.g., two independent reviewers) through each phase of the review (i.e., screening, eligibility, and inclusion in meta-analysis) | ✓ |  |  |
| Data collection process | 11c | Describe planned method of extracting data from reports (e.g., piloting forms, done independently, in duplicate), any processes for obtaining and confirming data from investigators | ✓ |  |  |
| **Data items** | 12 | List and define all variables for which data will be sought (e.g., PICO items, funding sources), any pre-planned data assumptions and simplifications | ✓ |  |  |
| **Outcomes and prioritization** | 13 | List and define all outcomes for which data will be sought, including prioritization of main and additional outcomes, with rationale | ✓ |  |  |
| **Risk of bias in individual studies** | 14 | Describe anticipated methods for assessing risk of bias of individual studies, including whether this will be done at the outcome or study level, or both; state how this information will be used in data synthesis | ✓ |  |  |
| ***DATA*** | | | | | |
| **Synthesis** | 15a | Describe criteria under which study data will be quantitatively synthesized | ✓ |  |  |
|  | 15b | If data are appropriate for quantitative synthesis, describe planned summary measures, methods of handling data, and methods of combining data from studies, including any planned exploration of consistency (e.g., *I* ^2^, Kendall’s tau) | ✓ |  |  |
|  | 15c | Describe any proposed additional analyses (e.g., sensitivity or subgroup analyses, meta-regression) | ✓ |  |  |
|  | 15d | If quantitative synthesis is not appropriate, describe the type of summary planned | ✓ |  |  |
| **Meta-bias(es)** | 16 | Specify any planned assessment of meta-bias(es) (e.g., publication bias across studies, selective reporting within studies) | ✓ |  |  |
| **Confidence in cumulative evidence** | 17 | Describe how the strength of the body of evidence will be assessed (e.g., GRADE) | ✓ |  |  |

**Appendix B in S1 File – Risk of Bias assessment scale** **[2]**

| Name of author(s): Year of publication:  Study title: | | | |
| --- | --- | --- | --- |
| **Risk of bias items** | | **Risk of bias levels** | **Points**  **scored** |
| 1. Was the study’s target population a **Yes** (**LOW RISK**): The study’s target population was a close 0  close representation of the national representation of the national population. population in relation to relevant  variables, e.g. age, sex, occupation?  **No** (**HIGH RISK**): The study’s target population was clearly NOT 1  representative of the national population. | | | |
| 2. | Was the sampling frame a true or close representation of the target population? | **Yes** (**LOW RISK**): The sampling frame was a true or close  representation of the target population. | 0 |
|  |  | **No** (**HIGH RISK**): The sampling frame was NOT a true or close  representation of the target population. | 1 |
| 3. Was some form of random selection used to select the sample, OR, was a census undertaken? | | **Yes** (**LOW RISK**): A census was undertaken, OR, some form of random 0  selection was used to select the sample (e.g. simple random sampling, stratified random sampling, cluster sampling, systematic sampling). | |
|  |  | **No** (**HIGH RISK**): A census was NOT undertaken, AND some form of  random selection was NOT used to select the sample. | 1 |
| 4. | Was the likelihood of non-response bias minimal? | **Yes** (**LOW RISK**): The response rate for the study was ≥75%, OR, an  analysis was performed that showed no significant difference in relevant demographic characteristics between responders and non- responders | 0 |
|  |  | **No** (**HIGH RISK**): The response rate was <75%, and if any analysis comparing responders and non-responders was done, it showed a significant difference in relevant demographic characteristics between  responders and non-responders | 1 |
| 5. | Were data collected directly from the  subjects (as opposed to a proxy)? | **Yes** (**LOW RISK**): All data were collected directly from the subjects. | 0 |
|  |  | **No** (**HIGH RISK**): In some instances, data were collected from a proxy. | 1 |
| 6. Was an acceptable case definition  used in the study? | | **Yes** (**LOW RISK**): An acceptable case definition was used.  **No** (**HIGH RISK**): An acceptable case definition was NOT used | 0  1 |
| 7. | Was the study instrument that measured the parameter of interest (e.g. prevalence of low back pain) shown to have reliability and validity (if necessary)? | **Yes** (**LOW RISK**): The study instrument had been shown to have  reliability and validity (if this was necessary), e.g. test-re- test, piloting, validation in a previous study, etc. | 0 |
|  |  | **No** (**HIGH RISK**): The study instrument had NOT been shown to have  reliability or validity (if this was necessary). | 1 |
| 8. | Was the same mode of data collection used for all subjects? | **Yes** (**LOW RISK**): The same mode of data collection was used for all  subjects. | 0 |
|  |  | **No** (**HIGH RISK**): The same mode of data collection was NOT used  for all subjects. | 1 |
| 9. Were the numerator(s) and denominator r(s) for the parameter of interest appropriate | | **Yes** (**LOW RISK**): The paper presented appropriate numerator(s) AND  denominator(s) for the parameter of interest (e.g. the prevalence of low back pain). | 0 |
|  |  | **No** (**HIGH RISK**): The paper did present numerator(s) AND  denominator(s) for the parameter of interest but one or more of these were inappropriate. | 1 |
| 10. Summary on the overall risk of study bias | | **LOW RISK** | 0-3 |
|  |  | **MODERATE RISK** | 4-6 |
|  |  | **HIGH RISK** | 7-9 |

Reference

1. Moher D, Shamseer L, Clarke M, Liberati A, Petticrew M, et al. Preferred reporting items for systematic review and meta-analysis protocols (PRISMA-P) 2015 statement. *Syst Rev* 4, 1(2015). <https://doi.org/10.1186/2046-4053-4-1>

2. Hoy D, Brooks P, Woolf A, Blyth F, March L, Bain C, et al. Assessing risk of bias in prevalence studies: modification of an existing tool and evidence of interrater agreement. J Clin Epidemiol. 2012 Sep;65(9):934-9. doi: 10.1016/j.jclinepi.2011.11.014. Epub 2012 Jun 27. PMID: 22742910.
